# Serum Therapy of Tetanus

**Published:** 1914-04

**Authors:** 


Serum Therapy of Tetanus. Jose Penna M. D., *La Semana Medica,* October 30, 1913, recommends the intravenous method of administration· He prefers it to lumbar puncture for, at times, the contracture of the vertebral column and the opisthotonos make the latter operation very difficult.

He believes that the antitoxin is of great benefit to the patient during the tetanic attack and that the nearer to the commencement of the attack the serum is given the greater the result.

When the tetanus is general in its most common clinical variety, the absorption has been affected by the peripheral nerves according to Meyer and Rawson, and to the blood stream by Gumprecht and Zupnik.

With reference to local tetanus, various theories prevail. That the toxin is fixed in the cells of the striated muscles; in the terminal ends of the motor nerves; in the terminal ends of the sensory nerves; in the ganglion cells of the spinal cord, reaching them by the nerve sheaths or by the axis-cylinders.

Experiments tend to prove that in ascending tetanus the toxin travels by the peripheral nerves, while in descending tetanus it is carried by the blood stream. This would account for the fact that in some cases the toxic agent is confined to a single segment in the medulla and in others the whole medulla is affected.

It has been found that when tetanus is provoked by an intravenous injection, there results an almost simultaneous contracture of all the muscles, but when increasing doses are given hypodermatically, the symptoms are partial primarily and general afterwards.

He believes that the tetanus toxin fixes itself in the protoplasm of the nerve cells of the cerebrum—the grey matter preferred. It is probable that the toxin contains at least two elements: A muscular poison and a hemolytic agent affecting the red blood cells.

Behring and Kitasata think that the blood of animals rendered immune by the antitoxin acquires the property of destroying the toxin.

For ten consecutive years Penna has used the intravenous method. He has increased the dosage until now he gives from 100-120cc of the serum at a time. No hard and fast rule can be laid down to determine the dose or the interval between doses in an individual case. The usual method is to inject 100cc into the veins of the patient every 12 to 24 hours during the first three days.

He concludes that the intravenous serum therapy in massive doses, frequently repeated, constitutes the ideal technique in tetanus.

